# Prevalence and risk factors for obstructive respiratory conditions among textile industry workers in Zimbabwe, 2006

**DOI:** 10.4314/pamj.v6i1.69063

**Published:** 2010-07-17

**Authors:** Mberikunashe Joseph, Banda Sarah, Chadambuka Addmore, Tafara Gombe Notion, Shambira Gerald, Tshimanga Mufuta, Matchaba-Hove Reginald

**Affiliations:** 1 Department of Community Medicine, University of Zimbabwe, P.O. Box A178, Avondale, Harare, Zimbabwe; 2 Ministry of Health and Child Welfare, Provincial Medical Directorate Mashonaland West, P.O. Box 139, Chinhoyi, Zimbabwe

**Keywords:** Respiratory obstruction, cotton dust, spirometry, Zimbabwe

## Abstract

**Introduction:** Workers in the cotton processing industries risk developing obstructive respiratory conditions due to prolonged exposure to cotton dust. We noted a tenfold increase in asthma among workers in a Textile Manufacturing Company. We determined the prevalence of respiratory obstructive conditions among workers in various sections. **Methods** We conducted a cross sectional analytic study. Workers were randomly sampled and data was collected using interviewer-administered questionnaires. Respiratory function was assessed using spirometry and chest auscultation. A walk through survey was conducted and a checklist was used to capture hazards and control measures in the work place. **Results** A total of 194 workers participated. The prevalence of severe respiratory obstruction was 27.8%. It was 50.0% among the blowers, 35.3% in waste recovery, 32.5% in carders, 15.0% in spinners and 7.5% among weavers. The mean years of exposure between the affected and the non-affected were significantly different (T =2.20; p< 0.05). Working in the blowing department was significantly associated with developing respiratory obstruction (OR=3.53; 95% CI= 1.61-7.79) but working in the weaving department was significantly protective (OR 0.16; CI 0.04-0.59).Working in a department for less than 10 years was protective (OR =0.94; 95% CI= 0.48-1.85), but not significant. **Conclusion** Obstructive respiratory conditions are common among textile workers, with those in blowing and waste recovery sections being the most affected. We recommended worker rotation every six months, regular spirometric screening employment of a medical officer.

## Introduction

A textile manufacturing company in Zimbabwe processes cotton to cloth at its two manufacturing plants. Cotton undergoes bale opening, blowing, carding, spinning and weaving. Bale opening (or blowing), carding and spinning is done at Plant A, whilst weaving is done at Plant B. The company employs around 1800 workers in their cotton mills.

According to a 1986 publication by the World Health Organization (WHO) on Pneumoconiosis and Smoking, workers in cotton processing industries risk developing obstructive respiratory conditions such as byssinosis and occupational asthma, due to prolonged exposure to inhalable cotton dust particles. Affected persons may be put on life-long treatment for asthma when in actual fact they do not have asthma, and all that may be required are occupational exposure control and safety measures [1]. In Zimbabwe little has been documented on the prevalence of byssinosis or other occupational lung diseases among cotton textile workers.

Records at the company clinic from December 2003 to December 2004 showed a tenfold increase (117 patients being followed up for asthma) in the number of cases recorded compared to an average of 11 to 15 per year before this period. We determined the prevalence of obstructive lung function pattern among textile workers, described the socio-demographic characteristics of the affected workers and determined factors associated with developing obstructive lung pathology. We assessed the workers’ knowledge with regard to health and safety issues in their working areas and identified potential hazards and control measures in the different work departments.

## Methods

An analytic cross sectional study was carried out at the textile manufacturing company’s two plants in Zimbabwe. Workers in the blowing, carding, spinning, weaving, and waste recovery and maintenance departments were selected from the company’s attendance registers using stratified random sampling. Each department was taken as a stratum. Workers were selected so that they proportionally represented workers in their department. Newly recruited workers (with less than 4 months at the company), and those on treatment for asthma were excluded from the study. Data was collected using a pre-tested interviewer-administered questionnaire, to assess the workers knowledge on potential risks in their working areas, safety issues and respiratory problems experienced by the worker. A separate questionnaire was used to interview key informants (nurses in charge of the company clinics, and the Health and Safety Officer), on the company’s policies on health and safety.

A walk through survey was conducted and a checklist was used to capture hazards and control measures in the work place. The workers’ respiratory functions were assessed using vitalograph spirometry tests, and chest auscultations were done by a medical doctor to determine the prevalence of respiratory obstruction. Spirometry test results were classified into severe obstruction (when FEV1 % < 65%), moderate obstruction (FEV1 % between 65% and 75%) and normal (FEV1% being 75% or more) [2]. Data was then captured and analyzed using Epi Info Version 3. 3. 2 statistical package to generate means, frequencies contingency tables and compute prevalence odds ratios at 95% significance level.

Informed verbal consent was obtained from all interviewees. All the information collected was treated as confidential. Workers found to be suffering from occupational related lung diseases were referred to the district hospital for appropriate management.

## Results

### Prevalence of Obstructive Pulmonary Function

A total of 194 workers were interviewed, and all were males. The median age of the study workers was 40 years (Q1= 31 years; Q3= 50 years), and the median years of experience in their current working areas was 12 years (Q1= 5 years, Q3= 21 years). Most of the workers, 131 out of 194 (67.5%), had attained secondary school education (Table 1).

Fifty-four out of 194 workers had severe respiratory obstruction, giving an overall prevalence of 27.8%. The prevalence of severe respiratory obstruction by departments were 50.0% for blowers, 35.3% waste recovery, 32.5% carders, 15.0% spinners, and 7.5% among weavers (Table 2). Using auscultation findings the overall prevalence of abnormal lung function was 44.4% (32.5% having wheezes, and 11.9% exhibiting reduced air entry), which is higher than that of severe lung obstruction as it included other lung abnormalities such as restrictive air entry patterns as well as obstructive ones. Wheezing was detected in 40.0% of blowers, 38.2% of those in waste recovery, 32.5% of spinners, 30.0% of carders, and 22.5% of the weavers. Reduced air entry was high among blowers and those in waste recovery (Table 2). There were 54 smokers among the study participants and of these 15 (27.78%) had severe obstruction, whilst 39 (27.86%) out of 140 non-smokers had severe obstruction. There was no significant difference between the two groups (χ²=0.03, p-value = 0.867).

### Factors associated with developing obstructed lung condition

Working in the blowing department was significantly associated with a higher risk of developing severe respiratory obstruction as compared to other departments [Odds Ratio (OR) =3.53; 95% CI 1.61-7.79]. Carding [OR=1.33; 95% CI 0.58-2.99] and waste recovery [OR=1.53; 95% CI 0.65-3.59] were also associated with a risk of developing obstructive lung pathology, though not statistically significant. Working in the weaving department was significantly protective [OR= 0.16; 95% CI 0.04-0.59]. Those exposed to working at the factory for less than 10 years were less likely to develop obstructive lung condition although this was not significant [OR=0.94; 95% CI 0.48-1.85]. No association was found between smoking and developing obstructive lung condition (OR=1.00; 95% CI 0.47-2.12) (Table 3). However among the smokers, those affected smoked a significantly higher number of cigarettes per day for significantly longer periods than the ones who had normal spirometry readings [T statistic = 2.44; p-value < 0.01].

Significant differences were found between the mean age and mean years of exposure in the current work area for the workers with normal lung spirometry when compared with those with FEV1% below 75% [T statistic = 2.38; p<0.02 for mean age difference; and T statistic=2.20; p< 0.05 for mean years of exposure]. Significant differences were also found between those with moderate obstruction and those with normal findings. Increased age and increased years of exposure to cotton dust were found to be associated with the risk of developing respiratory obstruction [T statistic = 2.97; p<0.01 for mean age difference; and T statistic = 2.85; p<0.01 for mean years of exposure].

### Workers knowledge and practices

Most workers (96.4%) knew that exposure to cotton dust posed a risk to their health. Out of 194 workers 153 (78.9%), knew at least one area with minimal protection from occupational hazards. Whilst most workers (80.9%) said they were medically examined on engagement for employment, only 16 (8.2%) had extensive respiratory examinations including chest radiography and spirometry tests. The rest had other non-chest related examinations, such as being screened for sexually transmitted infections. Most workers (95.4%) reported not being routinely sent for periodic medical check-ups, and 45.9% reported rotation of workers around the different work sections was not routine. Seventy-eight (40.2%) of the 194 workers interviewed had nothing to protect themselves from inhaling cotton dust. A marginal majority (53.1%) felt provision of personal protective clothing was the best way of safeguarding workers’ health followed by provision of food items such as milk and maheu reported by 11.0%. Maheu is a traditional brew made by mixing water, mealie meal and sugar and left to ferment overnight.

**Table 1:** Demographic Characteristics of the Workers Interviewed by Department, 2006

**Department**	**Mean Age in Years**	**Mean Years in Current Department**	**Level of Education Attained**			
			**None**	**Primary**	**Secondary**	**Tertiary**
Blowing (n=40)	43.6	18.2	0	14	26	0
Carding (n=40)	39.7	11.2	0	13	27	0
Spinning (n=37.3)	37.3	11.5	0	10	30	0
Waste recovery (n=34)	37.3	8.7	0	7	26	1
Weaving (n=40)	47.1	19.9	2	16	22	0
Total (n=194)	41.2	14.0	2	60	131	1

**Table 2:** The distribution of abnormal lung function amongst the workers at David Whitehead Textile Company, 2006

**Department**	**Spirometry Findings**		**Auscultation Findings**	
	**Severe obstruction** Number (%)	**Moderate obstruction** Number (%)	**Wheezing Number (%)**	**Reduced air entry** Number (%)
Blowing (n=40)	20 (50.0%)	7 (17.5%)	16 (40.0%)	7 (17.5%)
Carding (n=40)	13 (32.5%)	6 (15.0%)	12 (30.0%)	5 (12.5%)
Spinning (n=40)	6 (15.0%)	4 (10.0%)	13 (32.5%)	4 (10.0%)
Waste Recovery (n=34)	12 (35.3%)	5 (14.7%)	13 (38.2%)	6 (17.6%)
Weaving (n= 40)	3 (7.5%)	10 (25.0%)	9 (22.5%)	1 (2.5%)

**Table 3:** Factors associated with developing severe lung obstruction, David Whitehead Textile Company, Zimbabwe 2006

**Risk Factor**	**Deficient N=54**	**Normal N=140**	**Prevalence Odd ratios**	**95% Confidence Interval**
Blowing	20	20	3.53	1.61 – 7.79
Waste recovery	12	22	1.53	0.65 – 3.59
Carding	13	27	1.33	0.53 – 2.99
Spinning	6	34	0.39	0.14 – 1.06
Weaving	3	37	0.16	0.04 – 0.59*
Smoking	15	39	1.00	0.47 – 2.12
Working for less than 10 yrs	25	67	0.94	0.94 0.48 – 1.85

*Fisher’s exact

### Potential hazards and control measures

Major hazards identified were high cotton dust levels in most sections especially the Blow Room and Waste Recovery, noise from machines (Weaving Sheds, Speed Frames and Rings sections; these being names of the working areas), and unguarded moving machinery parts (Speed Frames, Waste Recovery and Weaving Sheds). Control measures found included the air conditioning system that was in place in most sections, which consisted of dust extracting fans and overhead fine-water spraying jets that push dust down. However, the ventilation system was either not working at all or was partially working in most departments. Out of the 103 workers seen at their workstations only 30 (29.1%) had plastic dust masks over their mouths and noses, Only one had a proper respirator on, and 3 had pieces of cloth as masks. All the workers had overalls, none had safety shoes on, 3 had goggles and 2 had ear plugs. The company had last distributed personal protection clothing three years prior to the study, citing foreign currency shortages. The company had one Health and Safety Officer but had no Health and Safety Policy Documents.

## Discussion

The prevalence of obstructive lung disease among the textile workers was about 28.0%, which was considerably high. This is an indication that prevention and control of cotton dust needs to be monitored. The trends of the respiratory abnormalities by departments however, are similar to findings in other studies done in Ethiopia, Egypt, South Africa and Sweden [3-5]. The study done in five Swedish cotton mills found that the prevalence of byssinosis was 19% among cotton workers, with males being more affected than females [3]. In the Ethiopian study, the prevalence of byssinosis was found to be 43.2% among blowers and 37.5% in carders, compared to 4-24% among workers in other sections. [4] In a similar study carried out in Alexandria, Egypt, the prevalence of byssinosis was 21.0% in bale opening sections and 13.0% in carding and combing rooms [5]. In a South African cotton textile industry the prevalence of byssinosis according to work departments was as follows: spinning 11.2%, winding 6.1%, and weaving 6.4% [6]. South Africa differs from other countries in having a high labour turnover rate, which reduces exposure periods. Their workers were found to have a high prevalence of previously treated pulmonary tuberculosis (3.4% for males and 2.2% for females), and in this population this disease appeared to cause more respiratory impairment than byssinosis [6]. The nature of cotton processing is such that more dust is created in the earlier processes and so the findings herein are not unexpected. The unexpected high prevalence of moderate lung obstruction among weavers (25.0%) could be due to the fact that workers in the weaving sheds often deal with ordinary cotton dust as well as dust from cotton dyed with different chemicals, thereby exposing them to chemical irritants.

The differences in the prevalence of lung pathologies as determined by spirometry tests and auscultation serves to show that occupational related lung conditions need a thorough, all encompassing medical examination to distinguish between temporary irritations and more serious lung pathology. The fact that most of the workers rarely had spirometry tests done on engagement and when they fall sick, could mean that a lot of serious lung pathologies could be going on unnoticed or were being treated as minor ailments.

Duration of exposure to cotton dust in the working area was found to be significantly associated with development of obstructive lung condition, with those who had worked in the same department for more than 10 years having a higher risk than those who had worked for less. This coupled with the finding that being aged above 40 years was associated with an increased risk for developing an obstructed lung pattern, especially in the spinning department, further supports the importance of routinely rotating workers and treating all work areas as risky. A case control study in Guangzhou, China found that the prevalence of byssinosis was higher in exposed workers, and that it increases with age, duration of exposure and cumulative inhalable dust exposure [7]. Another cohort study performed in Shanghai (1981-1996), showed that the prevalence of byssinosis increased over time in cotton workers, and that the long-term exposure to cotton dust is associated with chronic or permanent obstructive impairment [8].

Although smoking showed no significant association with one’s chance of developing cotton related lung obstruction, this could have been due to a small sample size of smokers recruited in this study than logical biological plausibility. This finding was in contrast with findings carried out in the Taiwan study where smoking increased the risk of cotton related lung disease [9].

The workers knowledge levels of occupational hazards that they were exposed to were high, but the fact that only 29.1% of the 103 workers found at their work areas, had their plastic dust masks on, and that the company last distributed personal protective clothing 3 years prior to the study, seemed to indicate a shortage of masks at the company. Lack of awareness on health and safety issues could also have resulted in workers having low risk perceptions, which caused them to shun personal protective gear. Personal protective measures are user dependent, can be uncomfortable and if not correctly used would fail to bring about the desired effect.

Although 157 (80.9%) of the workers said they had a pre-employment medical examination done, only 16 of these had chest radiography and vitalogragh spirometry tests done, with the majority being screened for such conditions as sexually transmitted infections, which are not quite relevant or related to the textile environment that the workers would be endeavoring to work in. With no regular or periodic medical check-ups being done the workers’ health is further compromised, and those at high risk would be missed and only caught when they are already sick.

The study design (cross-sectional) used in this study made it difficult for the temporal sequence of the exposure and the outcome of respiratory obstruction, as the exposure and outcome were measured at the same time. Another limitation of this study is that inhalable cotton dust levels in the different departments could not be measured due to the unavailability of the appropriate equipment for such an exercise.

## Conclusion

The prevalence of definite obstructive respiratory conditions at the textile manufacturing company was found to be high, with workers in the blowing and waste recovery sections having the highest risk. Workers aged above 40 years and those working in a dusty area continuously for more than 10 years had significantly high risk of developing obstructive respiratory pathology. The company’s health and safety policy needs to be developed and to put emphasis on regular medical check-ups for the workers, and incorporating training workshops for workers on occupational safety issues. We recommend regular sampling of air dust levels in the different work areas to ensure levels are below the permissible exposure limits.

## Competing interests

The authors declare no conflict of interest.

## Authors’ contributions

J Mberikunashe: He was responsible for the conception of the problem, design, collection, analysis and interpretation of data and drafting the final article. R Matchaba-Hove: He was responsible for the conception of the problem, design, analysis and interpretation of data and drafting the final article. S. Banda: She was responsible for the conception of the problem, design, collection, analysis and interpretation of data and drafting the final article. Mufuta Tshimanga: Had oversight of all the stages of the research and critically reviewed the final draft for academic content. Gerald Shambira: Had oversight of all the stages of the research and critically reviewed the final draft for academic content. Addmore Chadambuka: Participated in the design, analysis and interpretation of data and drafting the final article and critical review of the final draft. NT Gombe: He was responsible for the design, analysis and interpretation of data and drafting the final article.
